# Supplementary material for: Metabolomics Response to Drought Stress in Morus alba L. Variety Yu-711
Source: Plants (Basel). 2021 Aug 9;10(8):1636. doi: 10.3390/plants10081636 (PMC8400578; doi:10.3390/plants10081636)
Supplement: Supplementary file 1 [file plants-10-01636-s001.zip › plants-1303570 - supplementary for XML.pdf]

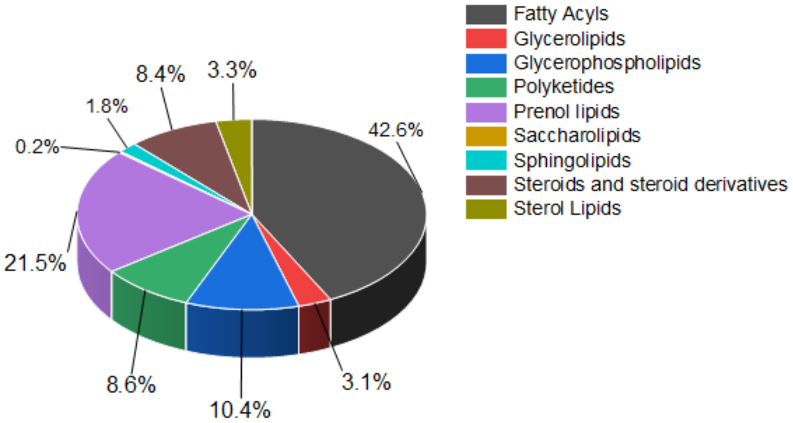

Figure S1. The proportion of lipids differentially expressed in mulberry Yu-711 under drought stress. After withholding water, Yu-711 leaves were harvested, and metabolites were analyzed by liquid chromatography-mass spectrometry (LC-MS). Each layer represents a class of lipids.

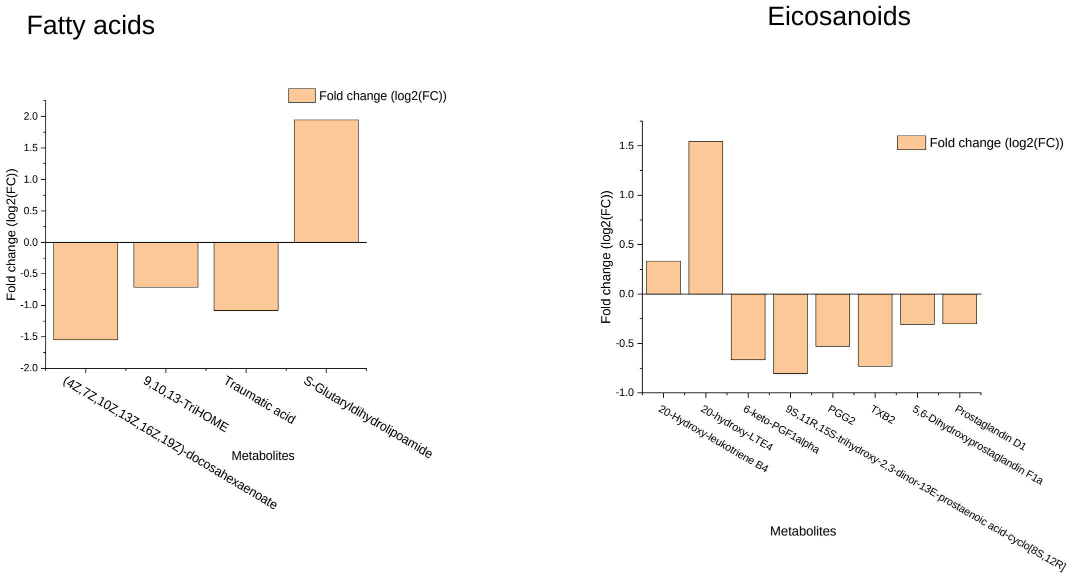

Linoleic acids

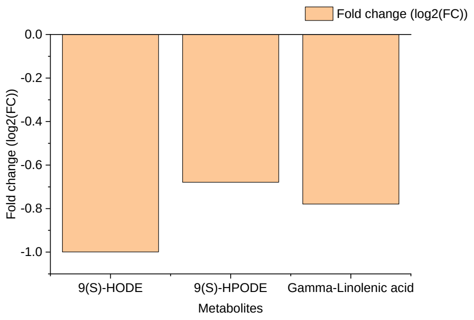

Others

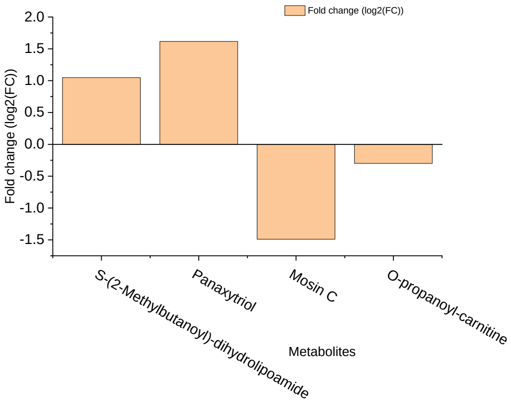

Figure S2. Fatty acyl lipid composition changes in Yu-711 plants under drought conditions. After withholding water, mulberry leaves were harvested, and the metabolites were analyzed using LC-MS. The vertical axis represents the fold change (FC) between the control and the drought-stressed plants. The horizontal axis represents each metabolite. Column pointing upwards represents an increase in FC, and column pointing downwards means a decrease in FC.

Terpenoids

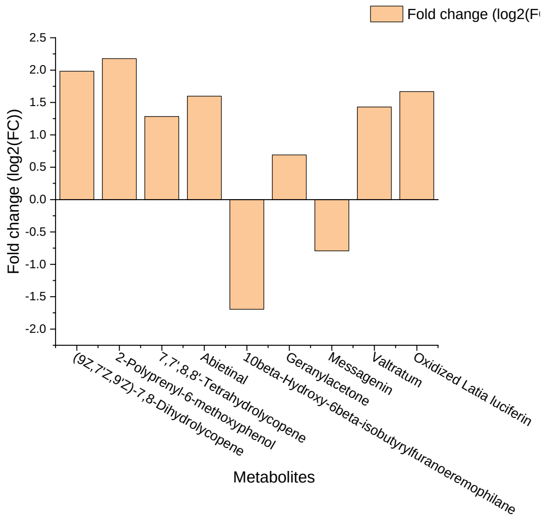

Isoprenoids

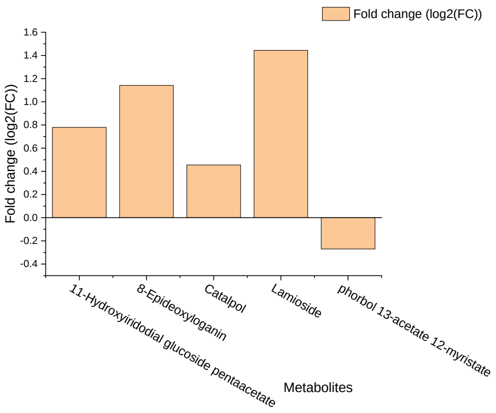

Others

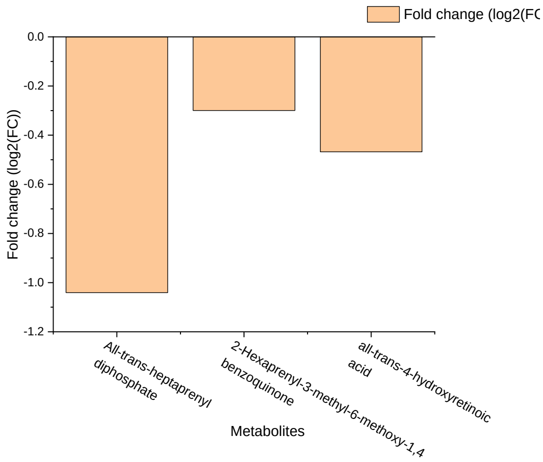

a

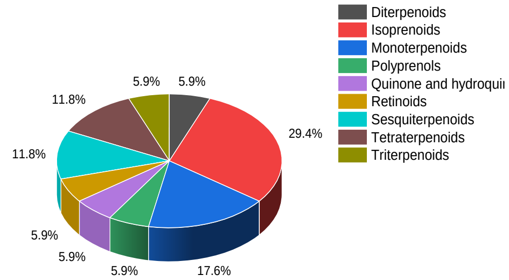

Figure S3. Prenol lipid composition changes in Yu-711 plants under drought conditions. After withholding water, mulberry leaves were harvested, and LC-MS analyzed the metabolites. The vertical axis represents the fold change (FC) between the control and the drought-stressed plants. The horizontal axis represents each metabolite. Column pointing upwards represents an increase in FC, and column pointing downwards means a decrease in FC. (**a**) the proportion of prenol lipids that changed significantly.

Glycerophospholipids

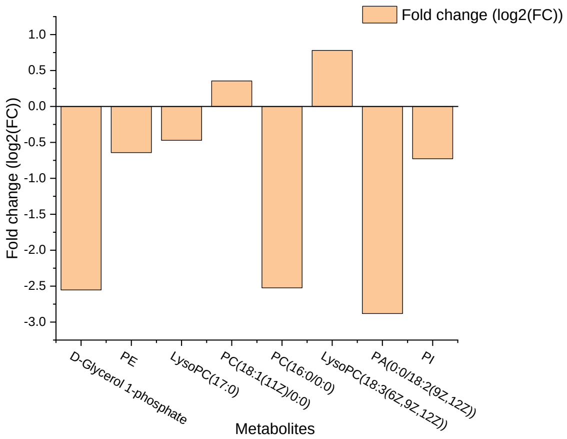

Polyketides

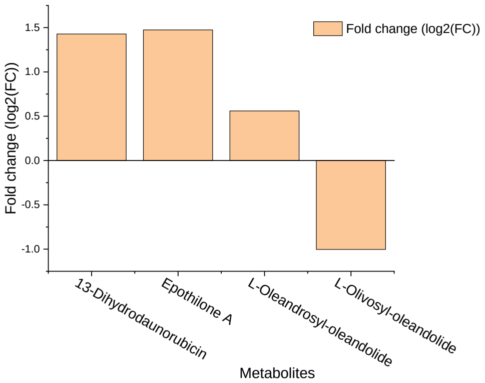

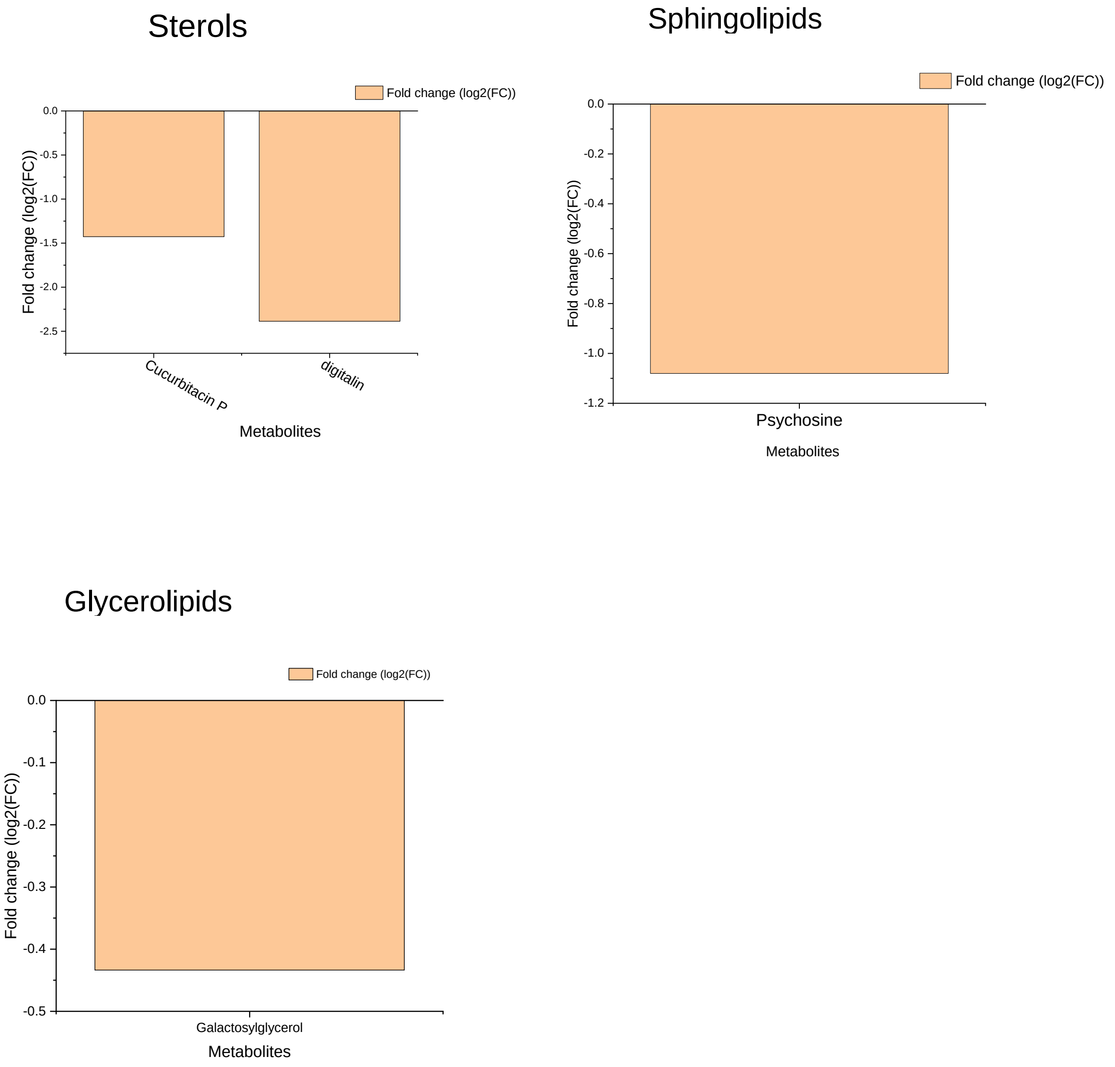

Figure S4. Other class of lipid composition changes in Yu-711 plants under drought conditions. After withholding water, mulberry leaves were harvested, and the metabolites were analyzed via LC-MS. The vertical axis represents the fold change (FC) between the control and the drought-stressed plants. The horizontal axis represents each metabolite. Column pointing upwards represents an increase in FC, and column pointing downwards means a decrease in FC. (abbreviations: PE, glycerylphosphorylethanolamine; LysoPC, lysoglycerophosphocholines; PC, glycerophosphocholines; PA, glycerophosphates; PI, glycerophosphoinositols.

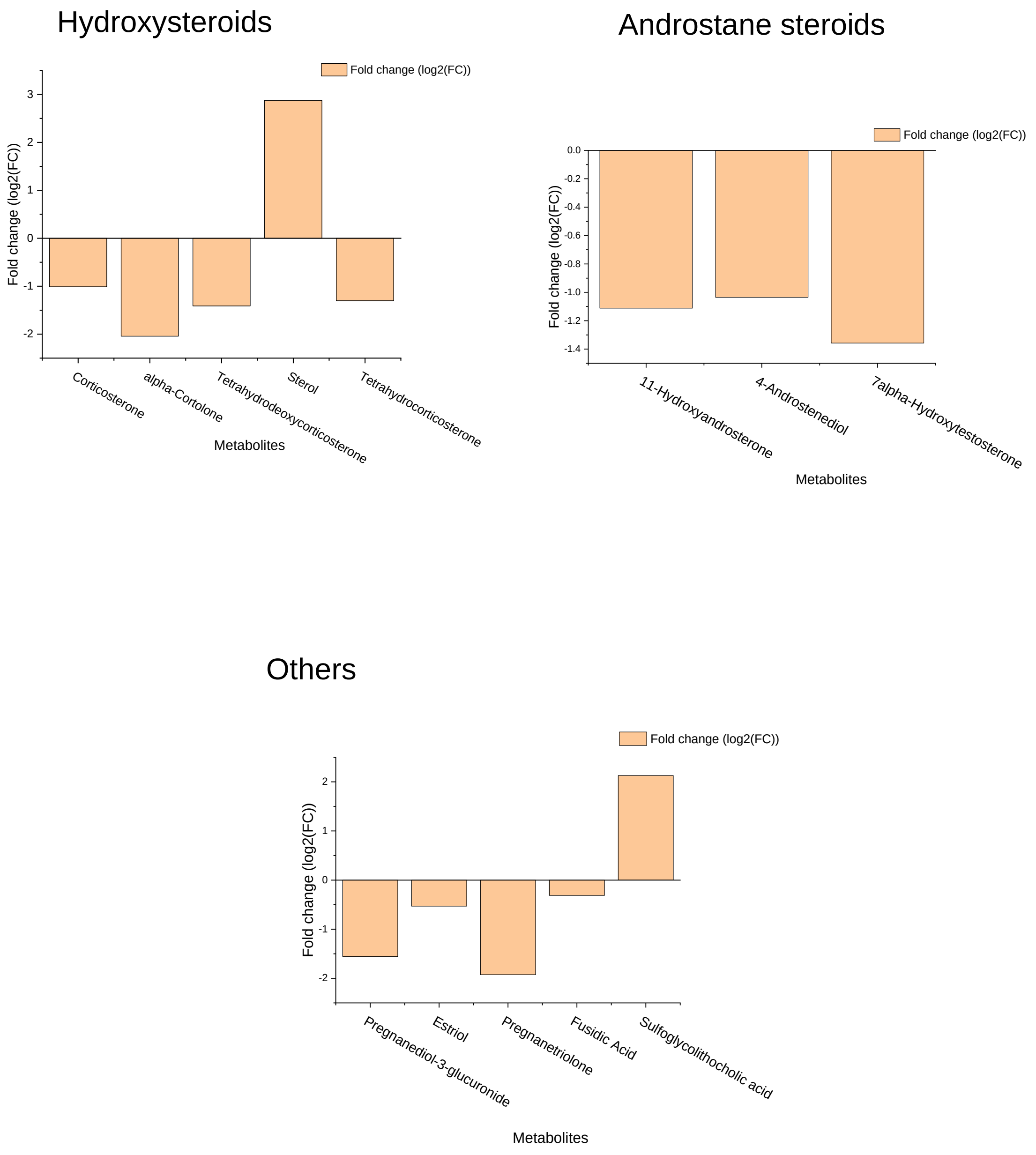

Figure S5. Steroid lipid composition changes in Yu-711 plants under drought conditions. After withholding water, mulberry leaves were harvested, and the metabolites were analyzed by LC-MS. The vertical axis represents the fold change (FC) between the control and the drought-stressed plants. The horizontal axis represents each metabolite. Column pointing upwards represents an increase in FC, and column pointing downwards means a decrease in FC.

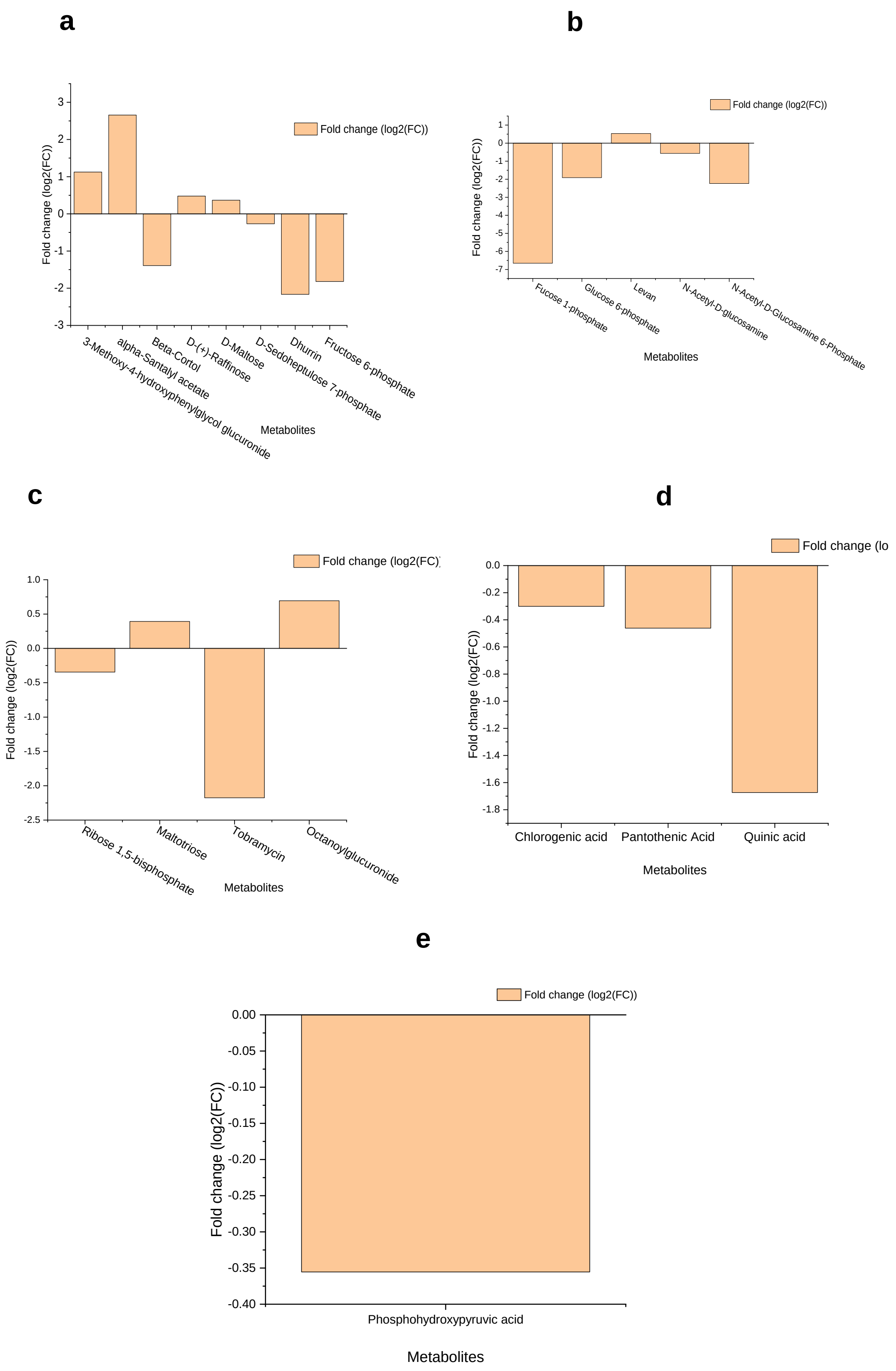

Figure S6. Organooxygen composition changes in Yu-711 plants under drought conditions. After withholding water, mulberry leaves were harvested, and the metabolites were analyzed by LC-MS. The vertical axis represents the fold change (FC) between the control and the drought-stressed plants. The horizontal axis represents each metabolite. Column pointing upwards represents an increase in FC, and column pointing downwards means a decrease in FC. (**a**–**c**) represents the class of carbohydrates composition changes. (**d**) represents alcohols and polyols class. (**e**) Is the carbonyl class.

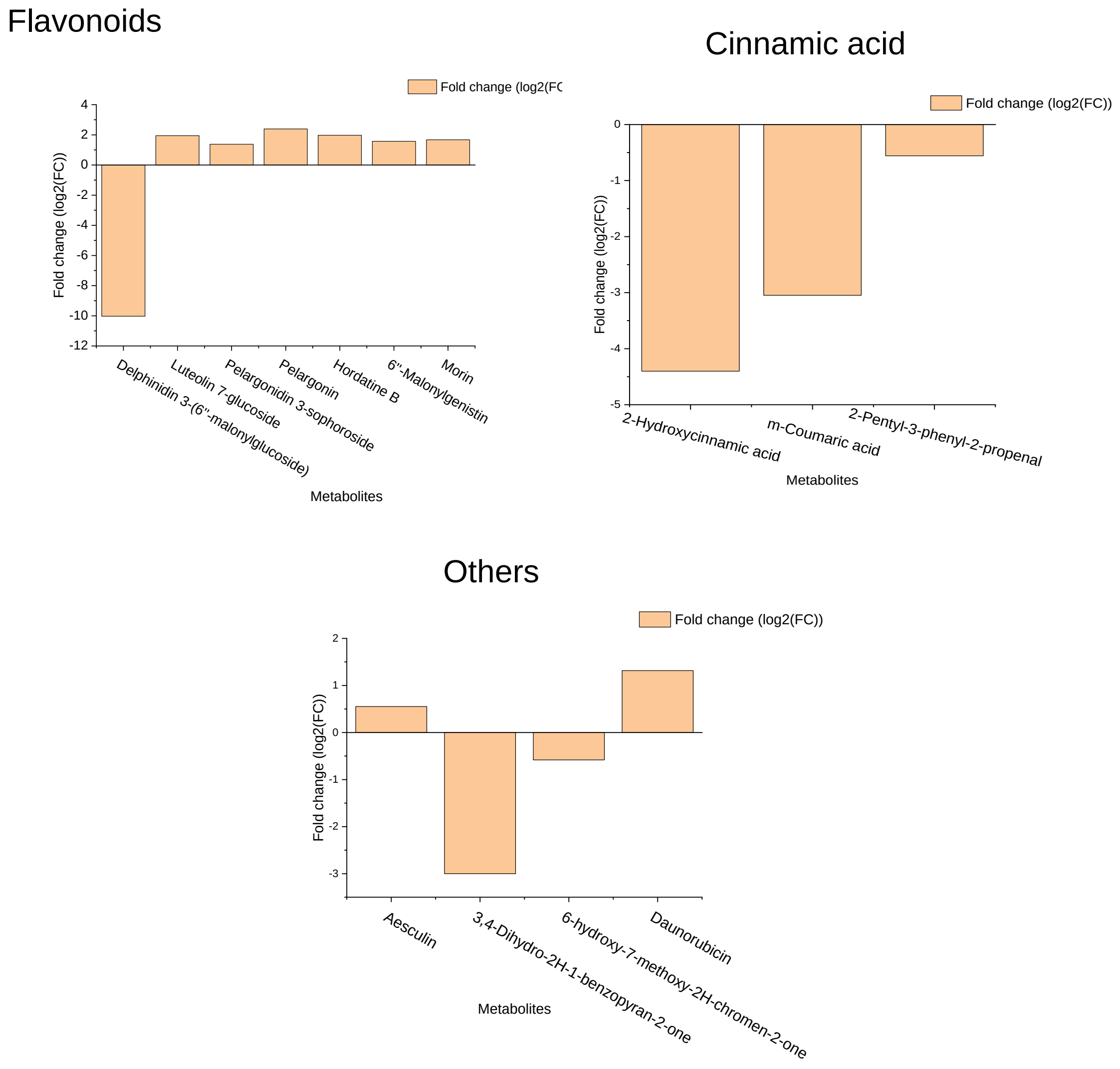

Figure S7. Phenylpropanoid composition changes in Yu-711 plants under drought conditions. After withholding water, mulberry leaves were harvested, and the metabolites were analyzed by LC-MS. The vertical axis represents the fold change (FC) between the control and the drought-stressed plants. The horizontal axis represents each metabolite. Column pointing upwards represents an increase in FC, and column pointing downwards means a decrease in FC.

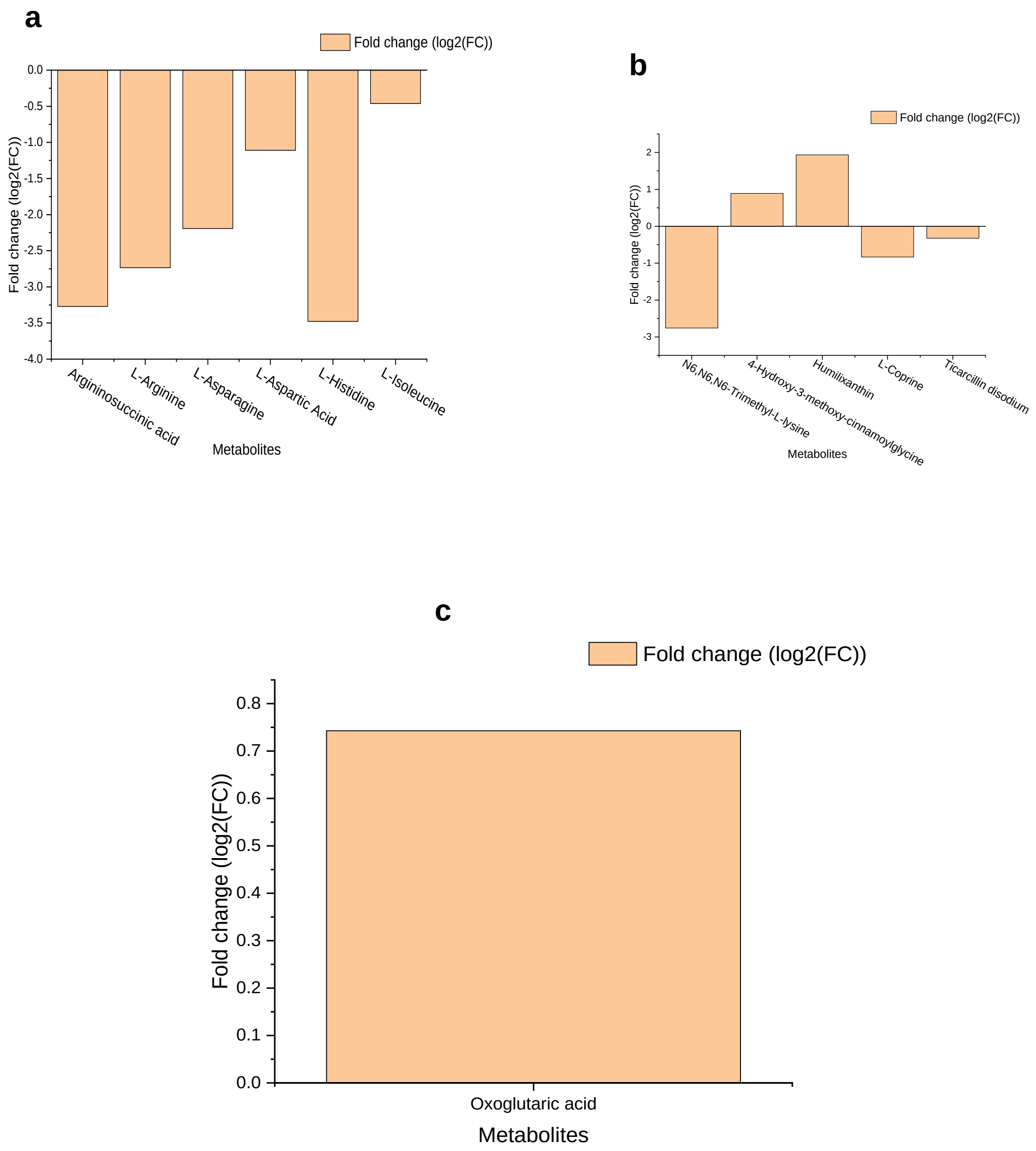

Figure S8. Organic acid composition changes in Yu-711 plants under drought conditions. After withholding water, mulberry leaves were harvested, and the metabolites were analyzed by LC-MS. The vertical axis represents the fold change (FC) between the control and the drought-stressed plants. The horizontal axis represents each metabolite. Column pointing upwards represents an increase in FC, and column pointing downwards means a decrease in FC. (**a**,**b**) represents the class of amino acid composition changes. (**c**) represent the class keto acid composition.

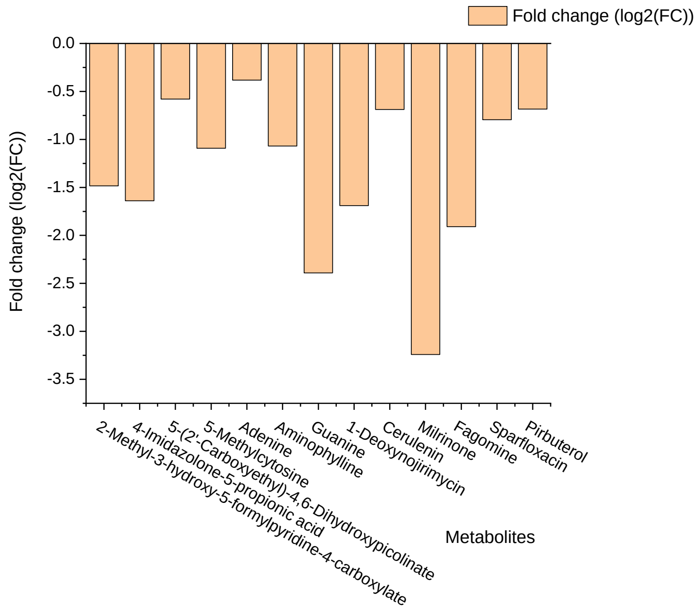

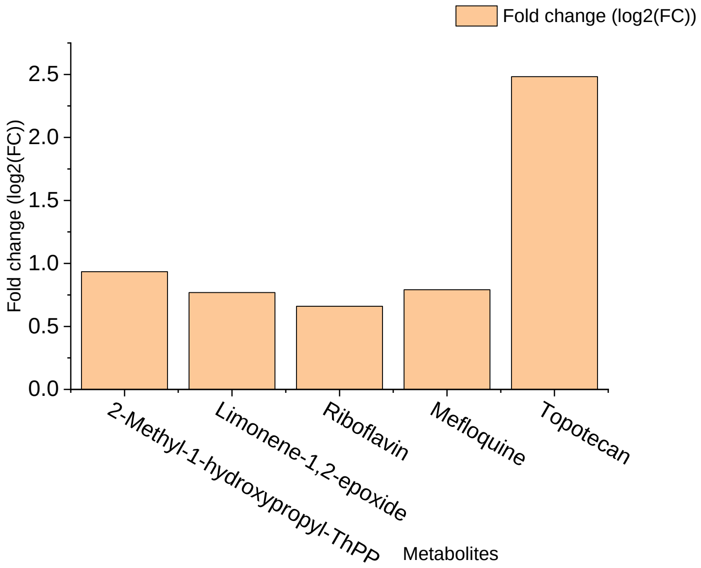

Figure S9. Organoheterocyclic composition changes in Yu-711 plants under drought conditions. After withholding water, mulberry leaves were harvested, and the metabolites were analyzed by LC-MS. The vertical axis represents the fold change (FC) between the control and the drought-stressed plants. The horizontal axis represents each metabolite. Column pointing upwards represents an increase in FC, and column pointing downwards means a decrease in FC.

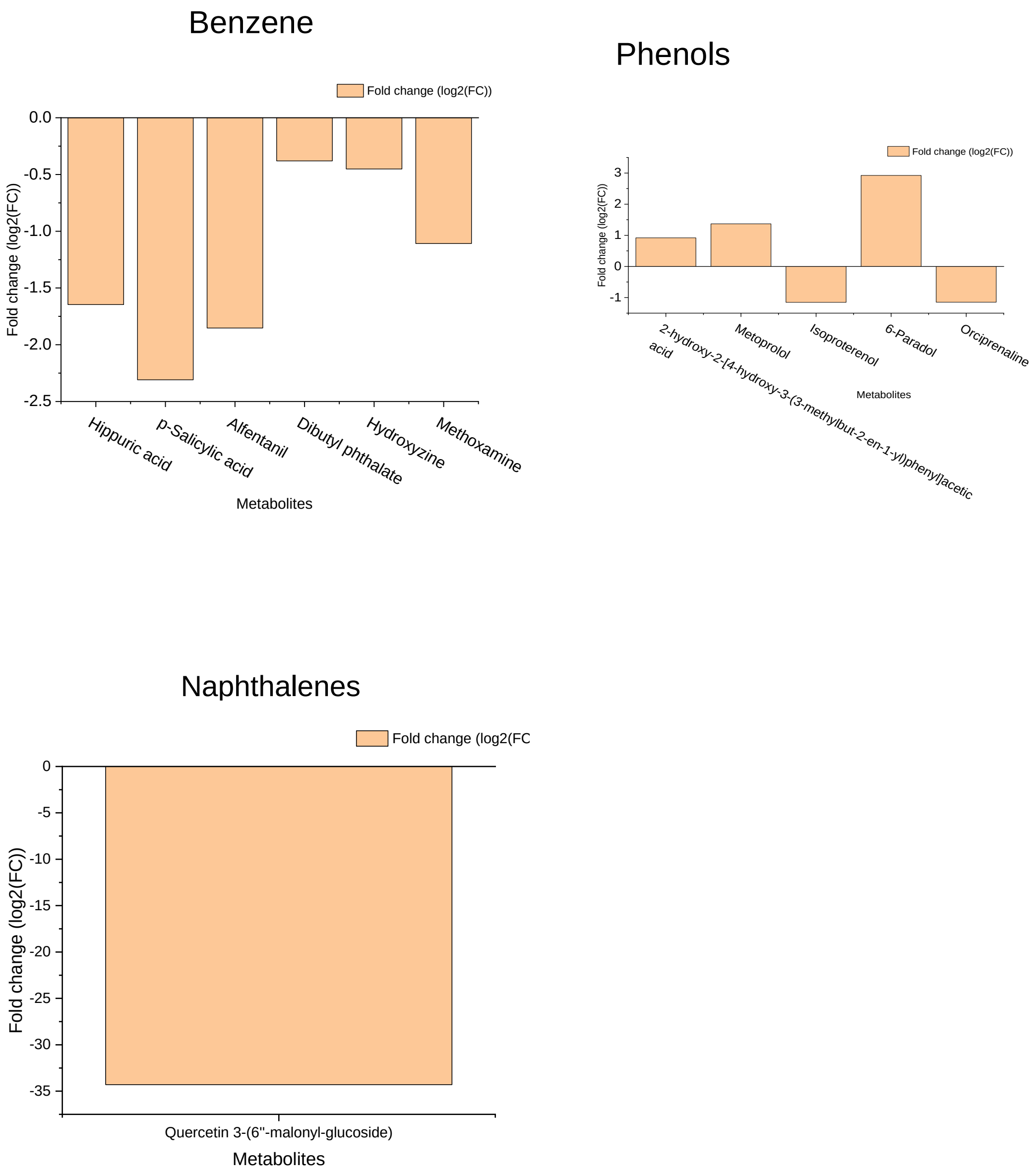

Figure S10. Benzenoid composition changes in Yu-711 plants under drought conditions. After withholding water, mulberry leaves were harvested, and the metabolites were analyzed by LC-MS. The vertical axis represents the fold change (FC) between the control and the drought-stressed plants. The horizontal axis represents each metabolite. Column pointing upwards represents an increase in FC, and column pointing downwards means a decrease in FC.

a. Arginine biosynthesis

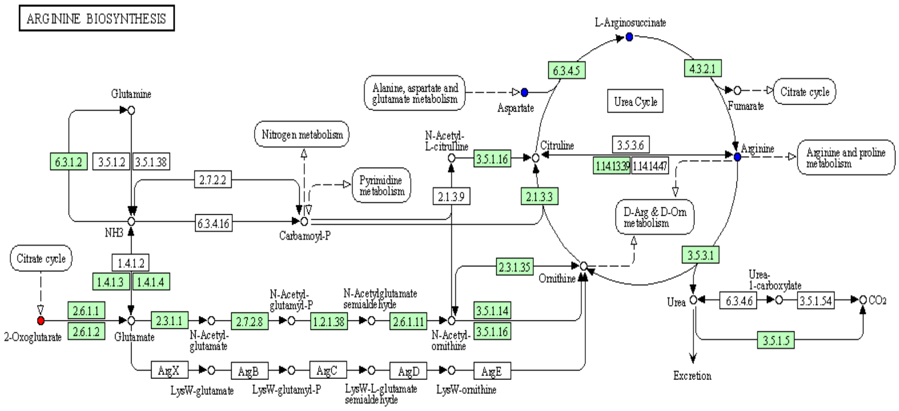

b. Galactose metabolism

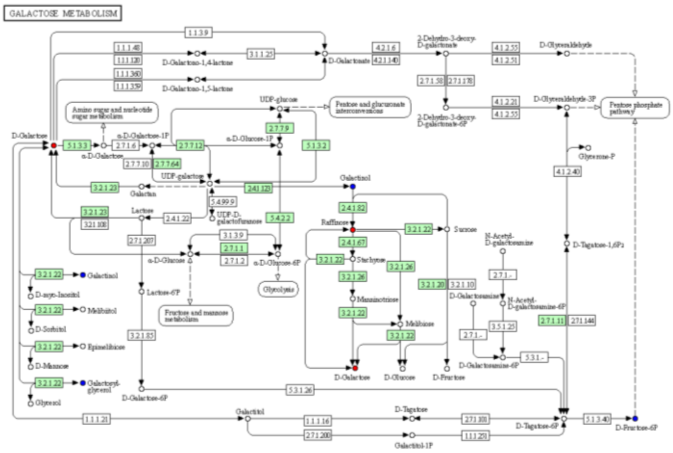

c. Alanine, aspartate and glutamate metabolism

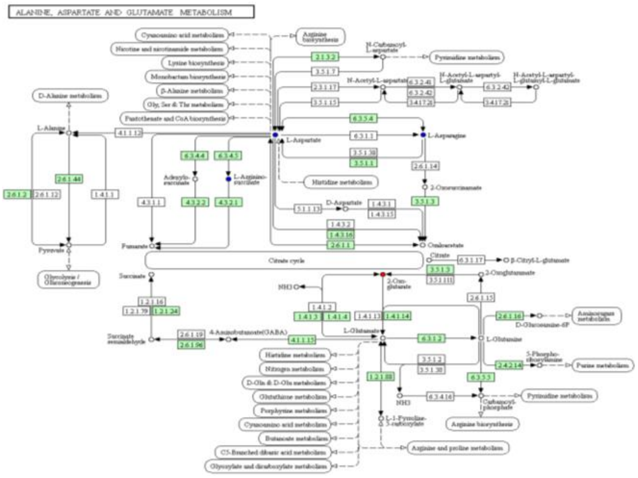

d. Linolenic acid metabolism

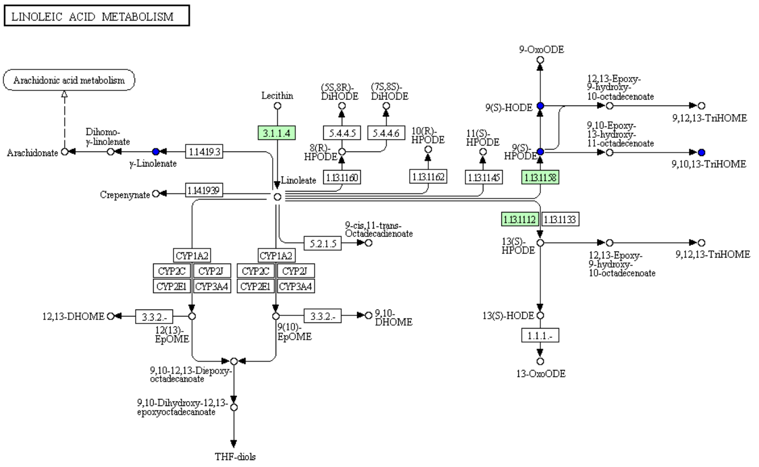

Figure S11. Metabolites pathway map of Arginine biosynthesis (**a**), Galactose metabolism (**b**), Alanine, aspartate and glutamate metabolism (**c**), Linolenic acid metabolism (**d**). The red circle represents up-regulated metabolites. The blue circle shows down-regulated metabolites.

a. ABC transporters

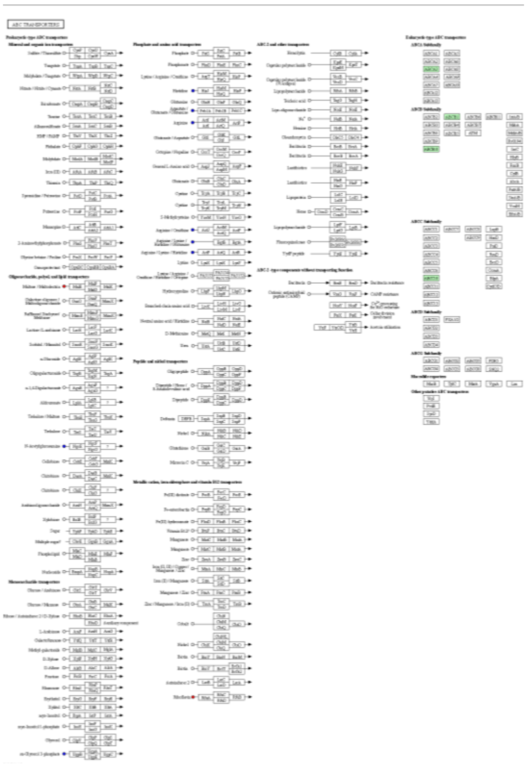

b. Aminoacyl-tRNA biosynthesis

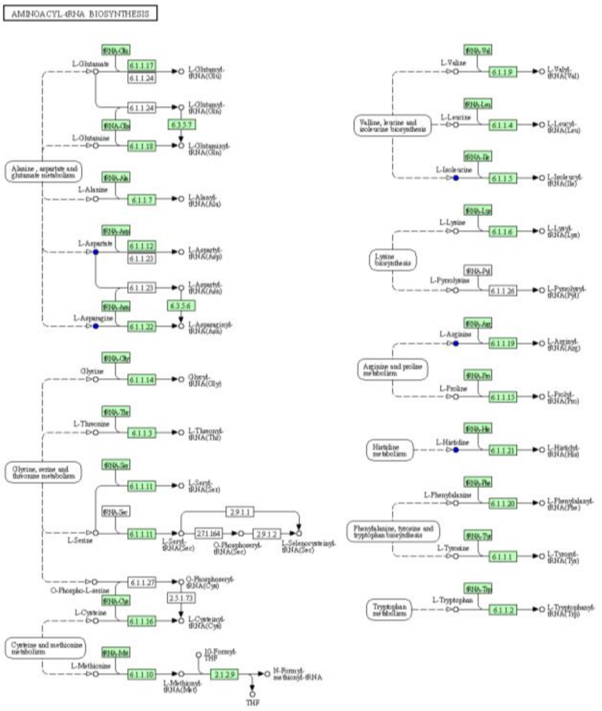

c. Arachidonic acid metabolism

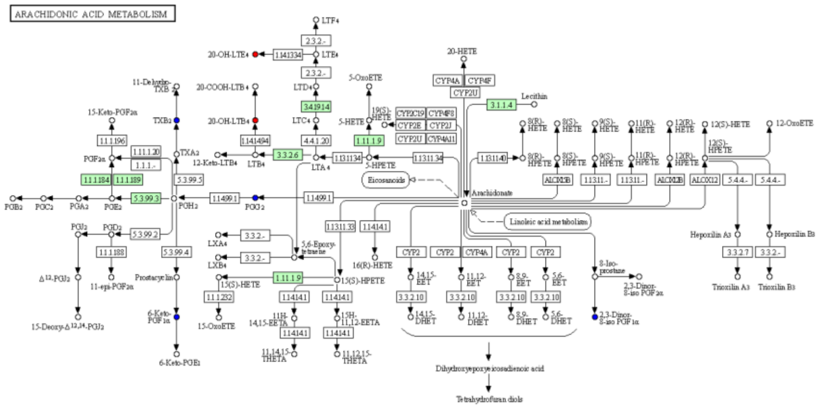

d. Glycerophospholipids metabolism

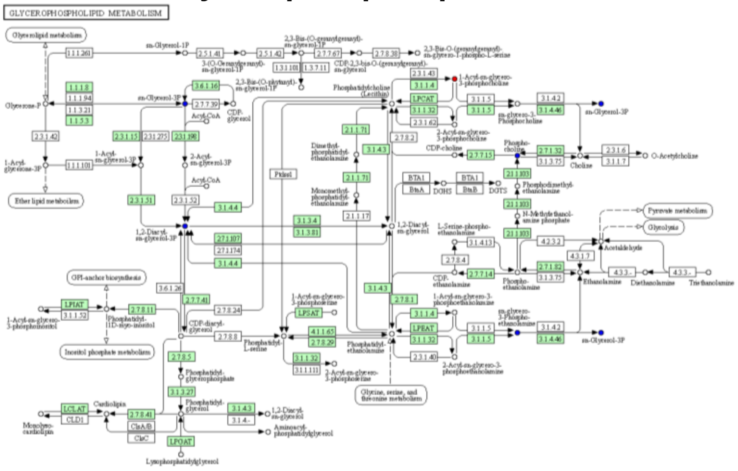

Figure S12. Metabolites pathway map of ABC transporters (**a**), Aminoacyl-tRNA biosynthesis (**b**), Arachidonic metabolism (**c**), glycerophospholipids metabolism (**d**). The red circle represents up-regulated metabolites. The blue circle shows down-regulated metabolites.

a. Carbon fixation in photosynthetic organisms

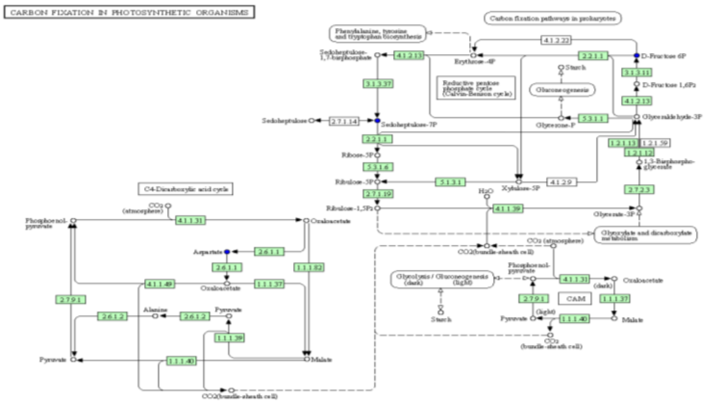

b. Cyanoamino acid metabolism

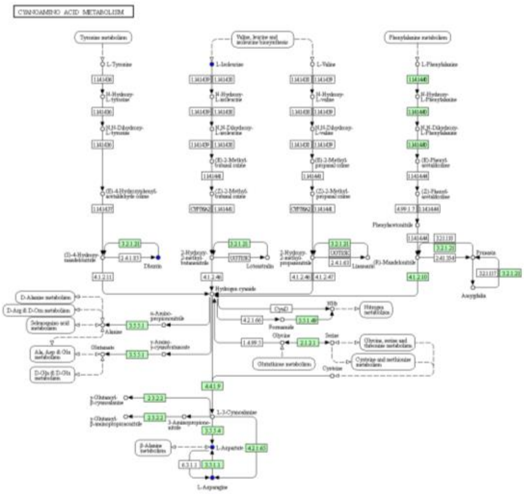

c. Histidine metabolism

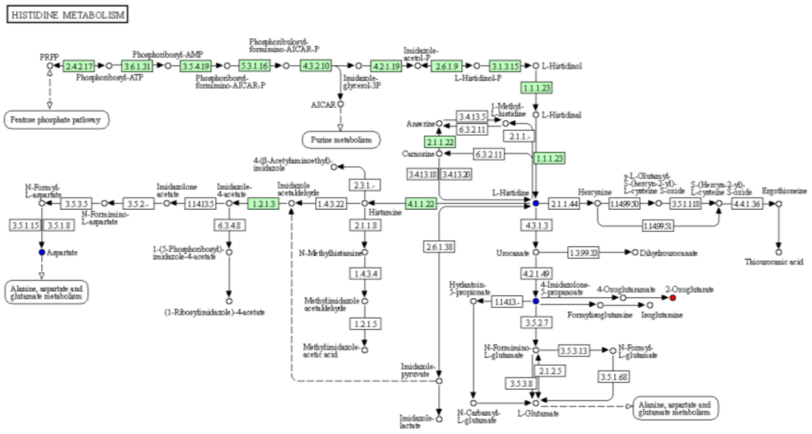

d. beta-alanine metabolism

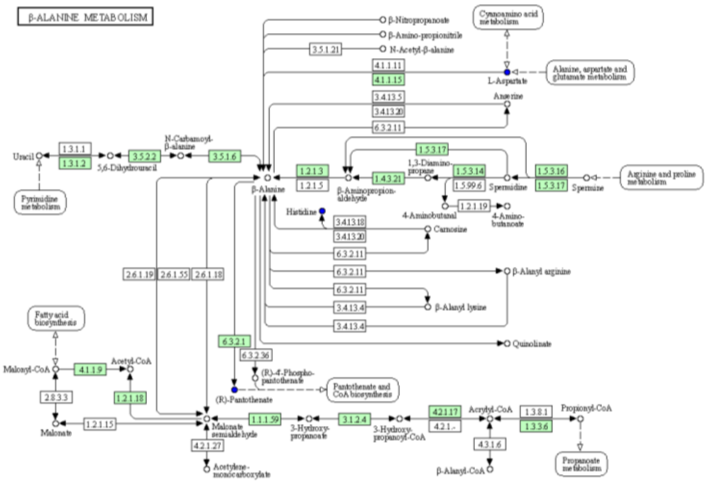

Figure S13. Metabolites pathway map of Carbon fixation photosynthesis organism (**a**), Cynoamino acid metabolism (**b**), Histidine metabolism (**c**), Beta-

alanine metabolism (**d**). The red circle represents up-regulated metabolites. The blue circle shows down-regulated metabolites.

a. Starch and sucrose metabolism

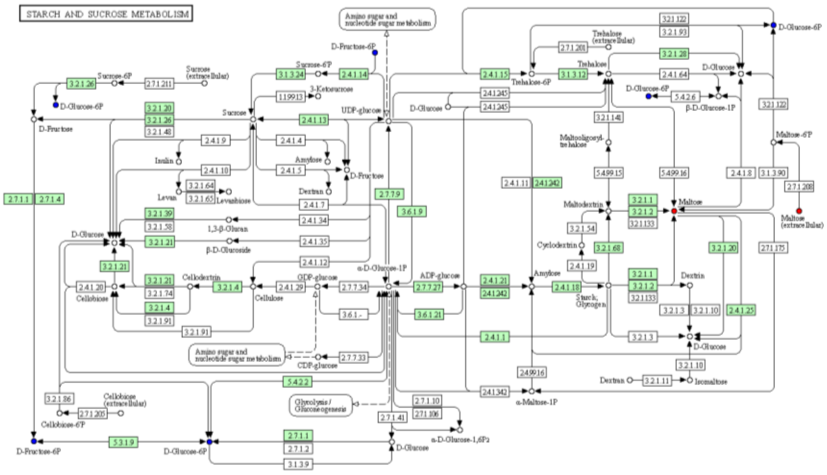

b. Glycerolipids metabolism

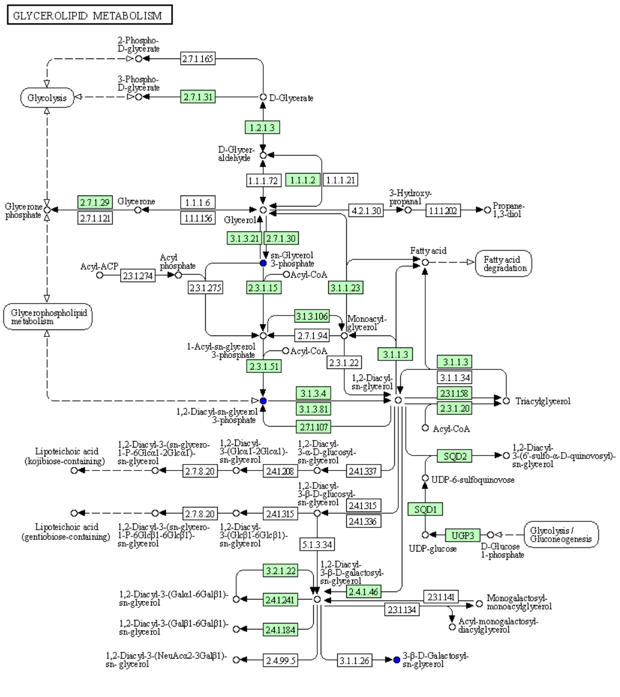

Figure S14. Metabolites pathway map of Starch and sucrose metabolism (**a**), Glycerolipids metabolism (**b**). The red circle indicates up-regulated metabolites, and the blue process represents down-regulated metabolites.
